# Knockdown of lncRNA BDNF-AS inhibited the progression of multiple myeloma by targeting the miR-125a/b-5p-BCL2 axis

**DOI:** 10.1186/s12979-021-00258-5

**Published:** 2022-01-03

**Authors:** Min Chu, Yingchao Fan, Liting Wu, Xiaoyan Ma, Jinfeng Sao, Yonghua Yao, Wenfang Zhuang, Cui Zhang

**Affiliations:** Medical laboratory, Shidong Hospital Affiliated to University of Shanghai For Science and Technology, 999 Shiguang Road, Yangpu District, Shanghai, 200438 China

**Keywords:** Multiple myeloma, BDNF-AS, miR-125a-5p, miR-125b-5p, Bcl-2, Proliferation

## Abstract

**Purpose:**

This study aimed to explore the role of long non-coding RNA (lncRNA) BDNF-AS in the progression of multiple myeloma (MM).

**Methods:**

The expression of BDNF-AS, miR-125a-5p, and miR-125b-5p in MM serum and cell lines were detected by quantitative reverse transcriptase PCR (qRT-PCR). The binding relationships between miR-125a/b-5p and BDNF-AS or Bcl-2 were predicted by Starbase and verified by luciferase reporter assay and RNA immunoprecipitation (RIP) assay. Cell proliferation was evaluated by Cell Counting Kit-8 (CCK-8) assay and 5-ethynyl-2′-deoxyuridine (EdU) staining assay. Cell migration was evaluated by wound healing assay. The expression levels of apoptosis-related proteins were evaluated by Western blot analysis. The role of BDNF-AS was also investigated in a xenograft tumor model in vivo.

**Results:**

BDNF-AS was significantly upregulated, while miR-125a-5p and miR-125b-5p were downregulated in MM serum and corresponding cancer cell lines. Knockdown of BDNF-AS effectively inhibited the proliferation and migration of MM.1S and U266 cells, and co-transfection of miR-125a-5p or miR-125b-5p inhibitor and sh-BDNF-AS enhanced cell proliferation and migration compared with that in sh-BDNF-AS group. Knockdown of miR-125a-5p or miR-125b-5p significantly enhanced the proliferation and migration of MM.1S and U266 cells, and co-transfection of sh-Bcl-2 and miR-125a/b-5p inhibitor inhibited cell proliferation compared with that in miR-125a/b-5p inhibitor group. Moreover, knockdown of BDNF-AS increased the expression levels of apoptosis-related proteins (cleaved caspase 3 and cleaved PARP), while knockdown of miR-125a-5p or miR-125b-5p reduced the expression levels of these apoptosis-related proteins compared with knockdown of BDNF-AS. Furthermore, knockdown of BDNF-AS effectively suppressed MM tumor growth in vivo.

**Conclusion:**

Our findings revealed that knockdown of BDNF-AS inhibited the progression of MM by targeting the miR-125a/b-5p-Bcl-2 axis, indicating that BDNF-AS might serve as a novel drug target for MM.

**Supplementary Information:**

The online version contains supplementary material available at 10.1186/s12979-021-00258-5.

## Introduction

Multiple myeloma (MM) is a hematologic malignancy caused by the excessive proliferation of plasma cells in bone marrows [1]. MM accounts for approximately 1% of all human cancers and 10% of all hematologic malignancies [2]. The median age of MM patients at the time of diagnosis is 70 years old, with 37% of them younger than 65 years old and 63% above 65 years old, and the incidence rate of this disease is higher in men than that in women [3]. Despite increasing understanding of the pathogenic mechanisms of MM and the discovery of new therapeutic targets, MM is still an incurable disease with a low 5-year overall survival rate [4]. Therefore, better understanding of the specific pathobiology during MM would provide novel insights into the diagnosis and treatment for MM.

Long non-coding RNAs (lncRNAs), a class of RNA transcripts larger than 200 nucleotides and lack protein-coding functions [5], play important roles in various pathogenic processes [6]. With the rapid development of high throughput sequencing technologies in recent years, increasing numbers of lncRNAs have been identified in the progression of MM. For example, NEAT1 is upregulated and regulates M2 macrophage polarization in MM by targeting miR-214 [7]. MALAT-1 is downregulated in the bone marrow mononuclear cells isolated from MM patients and elevates the expression levels of HMGB1 to inhibit tumor cell apoptosis via promoting autophagy [8]. Overexpression of H19 induces bortezomib resistance by targeting the miR-29b-3p/MCL-1 axis in MM [9]. HOXB-AS1 promotes the growth of MM cells by modulating the stability of FUT4 mRNA [10]. In addition, other lncRNAs including ANRIL, SNHG16, and LUCAT1 are also closely associated with the progression of MM [11–13]. The brain-derived neurotrophic factor antisense RNA (BDNF-AS) is a naturally conserved lncRNA (located at chromosome region 11p14.1 and with 2036 bp in length) and exerts essential functions in various human diseases [14–16]. A previous study found that BDNF-AS was abnormally up-regulated in MM, and its sensitivity and specificity were very high in discriminating MM from healthy donors [17]. In addition, Ai et al., revealed that inhibition of BDNF-AS using lentiviral shRNA silencing inhibited MM cell growth and angiogenesis in the bone marrow milieu in vivo [18]. These evidences confirmed the important role of BDNF-AS in MM. However, its underlying molecular mechanisms in MM remain unclear, which attracted us to focus on it.

MicroRNAs (miRNAs) are another class of non-coding RNAs with approximately 21–23 nucleotides in length [19]. MiRNAs have been identified as critical regulators in the pathophysiology of MM. Previous studies have identified many miRNAs, including miR-342, miR-363 [20], miR-767-5p [21], miR-145-3p [22], miR-13 [23], and miR-125a-5p [24], can promote or inhibit the occurrence and development of MM and are regarded as potential novel biomarkers [25]. Increasing studies demonstrated that lncRNAs function as direct sponges for miRNAs to exert their essential biological functions in eukaryotic cells [26]. One previous study found that BDNF-AS enhances SH-SY5Y cell viability and inhibits autophagy and apoptosis in MPTP-induced Parkinson’s disease by suppressing miR-125b-5p [27], suggesting that miR-125b-5p is a target of BDNF-AS. MiR-125a-5p and miR-125b-5p are two important regulators of miR-125 family [28]. Therefore, we hypothesized that BDNF-AS might regulate MM progression by sponging miR-125b-5p and miR-125a-5p.

Previous studies have demonstrated that apoptosis plays a key role in protecting tissue homeostasis and humoral immune response [29]. Bcl-2 belongs to the Bcl-2 protein family and is an anti-apoptotic factor [30]. The anti-apoptotic function of Bcl-2 in different types of human cancer has been well studied, and targeting of Bcl-2 may be a novel modality of cancer therapy [31, 32]. Increasing studies have found that overexpression of Bcl-2 is a hallmark of cancer and contributes to tumor cell survival as well as the resistance to therapy in MM [33]. These reports suggest that downregulation of Bcl-2 and other molecules that can downregulate Bcl-2 might be potential therapeutic targets for MM. Moreover, Bcl-2 is a direct target of miRNAs to participate in the development of human cancers, such as miR-15 in thyroid cancer [34], miR-448 in hepatocellular carcinoma [35], and miR-1915-3p in gastric cancer [36].

This study revealed that knockdown of BDNF-AS effectively inhibited proliferation and promoted apoptosis of MM cells in vitro and suppressed tumor growth in vivo. Moreover, BDNF-AS positively regulated the expression of Bcl-2 by directly sponging miR-125a/b-5p. Overall, our results suggested that BDNF-AS might be a potential diagnostic and therapeutic target for MM.

## Materials and methods

### Patients and sample collection

A total of 30 MM patients (14 males and 16 females) and 30 healthy donors (15 males and 15 females) were recruited at the Shidong Hospital Affiliated to University of Shanghai for Science and Technology between November 2017 and August 2019. Written informed consent was obtained from all participants. This study was approved by the Human Ethics Committee of the aforementioned hospital and conducted in accordance with the Declaration of Helsinki (2000). The serum samples were collected from MM patients and healthy donors, then the supernatant serum was collected through centrifugation at 3000 g for 15 min, followed by transferring into an RNase-free Eppendorf tube and stored in − 80 °C for future use. All patients did not receive any treatment before sample collection. The expression of BDNF-AS and clinicopathological characteristics of MM patients were shown in Supplementary Table 1.

### Cell culture

Four MM cell lines (ARP-1, MM.1S, U266, and NCI-H929), normal plasma cell line (nPC), and HEK-293 T cells were purchased from the American Type Culture Collection (ATCC, Manassas, VA, USA). All cells were cultured in RPMI-1640 medium (GIBCO, Life Technologies, Carlsbad, CA, USA) supplemented with 10% fetal bovine serum (FBS; GIBCO) and 100 U/ml penicillin/streptomycin (GIBCO) at 37 °C in a humidified incubator with 5% CO_2_.

### Lentivirus transduction and cell transfection

The lentiviral vector carrying short hairpin RNA targeting BDNF-AS (sh-BDNF-AS), short hairpin RNA targeting Bcl-2 (sh-Bcl-2), and negative control (sh-NC) were designed and synthesized by GenePharma Co., Ltd. (Shanghai, China). The miR-125a-5p mimics, miR-125b-5p mimics, miR-125a-5p inhibitor, miR-125b-5p inhibitor, and their corresponding negative controls (miR-NC and inhibitor NC) were purchased from Ribobio (Guangzhou, China). Approximately 1.5 × 10^7^ viral particles and 100 pM miRNA mimics or inhibitor were transfected into HEK-293 T, MM.1S, and U266 cells using Lipofectamine 2000 (Thermo Fisher Scientific). After 48 h of transfection, puromycin (Sigma, USA) was added to screen HEK-293 T, MM.1S and U266 cells stably transfected with lentiviruses carrying sh-BDNF-AS, sh-Bcl-2, or sh-NC. The transfection efficiency was confirmed by qRT-PCR. The sequences of oligos used in this study were as follows: 5′-UCCCUGAGACCCUAACUUGUGA-3′ for miR-125b-5p mimic, 5′-TCACAAGTTAGGGTCTCAGGGA-3′ for miR-125b-5p inhibitor, 5′-UCCCUGAGACCCUUUAACCUGUGA-3′ for miR-125a-5p mimic, 5′-UCACAGGUUAAAGGGUCUCAGGGA-3′ for miR-125a-5p inhibitor, 5′-UUCUCCGAACGUGUCACGUTT-3′ for miR-NC, 5′-CAGUACUUUUGUGUAGUACAA-3′ for inhibitor-NC, 5′-GGCTCACCAGTTGTTTGTT-3′ for sh-BDNF-AS, and 5′-UAAGGCUAUGAAGAGA UAC-3′ for sh-NC.

### RNA isolation and qRT-PCR

Total RNAs were extracted using TRIzol reagent (Thermo Fisher, USA) and reversely transcribed into complementary DNA (cDNA) using a PrimeScript Reverse Transcription Reagent kit (Takara, Beijing, China). Then qRT-PCR reactions were prepared using a SYBR Green PCR kit (Applied Biosystems) and performed on an ABI Prism 7500 system (Applied Biosystems). The relative expression levels of genes were calculated using the 2^-ΔΔCt^ method with GAPDH and U6 as the internal reference for lncRNA, Bcl-2 and miRNA, respectively. The sequences of primers used in this study were as follows: miR-125a-5p forward 5′-ACACTCCAGCTGGGC AGCAGCACACTGTGG-3′ and reverse: 5′-TGGTGTCGTGGAGTCG-3′, miR-125b-5p forward 5′-ACACTCCAGCTGGGCAGCAGCACACTGTGG-3′ and reverse 5′-TG GTGTCGTGGAGTCG-3′, U6 forward 5′-TGCGGGTGCTCGCTTCGGCAGC-3′ and reverse 5′-CCAGTGCAGGGTCCGAGGT-3′, BDNF-AS forward 5′-TTGATGGCTT GAACATTTGG-3′ and reverse 5′-TCGTGATT TTCGGTGTCTGT-3′, and GAPDH forward 5′-GAGTCAACGATTTGGTCGT-3′ and reverse 5′-GACAAGCTTCCCGTT CTCAG-3′.

### Western blot analysis

Total proteins were extracted using RIPA lysis buffer. Protein concentration was determined using a BCA Protein Assay Kit (Tiangen, Beijing, China). Equal amount of protein samples were separated by 10% SDS-PAGE and transferred onto PVDF membranes (Millipore, USA). After blocking in 3% BSA, the membranes were incubated with specific primary antibodies against cleaved caspase 3, total caspase 3, cleaved PARP, total PARP, Bcl-2, and GAPDH (all from Abcam, MA, USA) at 4 °C overnight. After washing TBST for 3 times, the membranes were incubated with HRP-conjugated secondary antibody at room temperature for 2 h. Protein bands were visualized using an enhanced chemiluminescence (ECL) kit (Millipore, Billerica, MA, USA), and the band intensity was analyzed using ImageJ software (National Institutes of Health, Bethesda, MD).

### Cell counting Kit-8 (CCK-8) assay

Cell viability was detected using Cell Counting Kit-8 Reagent (CCK-8, Dojindo, Japan). In brief, the transfected MM.1S and U266 cells were plated into a 96-well plate and cultured for 24, 48, 72, and 96 h. Then 10 μl CCK8 solution was added into each well and incubated for another 4 h. Finally, the absorbance at 450 nm was measured using a microplate reader.

### Luciferase reporter assay

The binding sites between miR-125a/b-5p, or miR-125a/b-5p and Bcl-2 were predicted by Starbase 3.0 software (http://starbase.sysu.edu.cn/). To determine their relationship, the entire Bcl2 3′-UTR (NM_000657.3) containing the wild-type (WT) or mutated (MUT) putative miR-125a/b-5p binding site were amplified and cloned into the XbaI-FseI sites of pGL3-Promoter Luciferase Expression Vector (Promega) and named Bcl-2-WT or Bcl-2-MUT, respectively. The primers were as follows: Bcl-2: forward 5′-GATCTCTAGAACATGCCTGCCCCAAACAAATATG-3′, reverse 5′-GATCTCTAGAACAGACAAGGAAAGTTTAATGGCAATGTG-3′, and mutation of the miR-125b-5p (position 2419–2426, Fig. 6A) and miR-125a-5p (position 2419–2426, Fig. 6A) binding sites was carried out using the QuikChange II Site-directed Mutagenesis kit (Agilent Technologies UK Ltd., Wokingham, UK). These luciferase reporter plasmids were co-transfected with miR-125a/b-5p mimics or miR-NC into HEK-293 T cells using Lipofectamine 2000. After transfection for 48 h, cells were lysed, and the relative luciferase activities were detected using the Dual Luciferase Reporter Assay system (Promega).

### Stoichiometric quantitation of BDNF and miR-125a/b-5p by RT-qPCR and droplet digital PCR analysis (ddPCR)

DDPCR was performed to quantify the absolute RNA copy numbers of BDNF-AS and miR-125a/b-5p in MM1S and U266 cells. In brief, 1 μg total RNA samples were reversely transcribed using a First-Strand cDNA Synthesis Kit. The droplets were generated using the QX200™ AutoDG™ Droplet Digital™ PCR System. The PCR reactions using the droplets were prepared using EvaGreen Supermix (1,864,033, Bio-Rad) containing 1 μl of cDNA. The absolute RNA copy numbers were assessed using QX200 Droplet Digital PCR System and calculated as previously described [37]. The copy numbers per cell were further estimated using a reference mRNA of known abundance as previously described [38].

### RNA immunoprecipitation (RIP) assay

RIP was performed using the EZ-Magna RIP Kit (Millipore, Billerica, MA, USA) following the manufacturer’s instructions. Briefly, HEK-293 T cells were lysed using RIP lysis buffer, and cell lysates were incubated with magnetic beads conjugated with anti-Ago2 antibody or negative control IgG (Millipore, USA). The immune-precipitated RNAs were then extracted, and the enriched binding targets were detected by qRT-PCR.

### EdU staining assay

Cell proliferation was evaluated using EdU (5-ethynyl-2′-deoxyuridine) staining assay as previously described [39]. In brief, the transfected MM.1S and U266 cells were seeded into 96-well plates. At 24 h after seeding, 100 μl fresh medium containing 50 μM EdU (Ribobio) was added into each well. Cells were then incubated for another 2 h, fixed with 4% paraformaldehyde for 20 min, and then counterstained with DAPI solution. The images were observed under a fluorescence microscope (Nikon) (× 200 magnification) and merged by Adobe Photoshop 6.0 software. The proliferation rate of cells was counted as the percentage of EdU positive cells in 5 random fields.

### Wound healing assay

Cell migration ability was examined as previously described [40]. In brief, cells were incubated with a normal cell growth medium in 6-well plates. The cell layers at approximately 90% adherence were scratched with a 10 μl sterile pipette tip. The shredded cells were washed with sterile PBS. After 24 h, images of cells in plates were acquired using a microscope and analyzed using Image J software. Cell migration rate was calculated as the percentage of the total cell-free area.

### Xenograft tumor model

A total of 8 BALB/c nude mice (male, 5–6 weeks old, approximately 18–20 g) were purchase from Beijing Vital River Laboratory Animal Technology Co., Ltd. (Beijing, China). All animal procedures were performed following the guidelines of the Institutional Animal Care and Use Committee (Permission No: 655). This study was approved by the Ethics Committee of Shidong Hospital Affiliated to University of Shanghai for Science and Technology. A xenograft tumor model with MM was established as previously described [41]. In brief, mice were randomly assigned into 2 groups with 4 mice in each group and injected subcutaneously with 1 × 10^6^ MM.1S cells stably transfected sh-BDNF-AS or sh-NC. The tumor volume was evaluated every week for 4 weeks uisng the formula: V = (L× W^2^)/2, where L is the tumor length and W is the tumor width. After 4 weeks, mice were sacrificed by cervical dislocation and the xenograft tumors were excised and weighed. The xenograft tumors were stored at − 80 °C for subsequent experiments.

### Immunohistochemistry (IHC) analysis

The xenograft tumors were embedded in paraffin and cut into 4-μm thick sections. The sections were then dewaxed with xylene, hydrated with gradient ethanol, and incubated with primary anti-Ki67 antibody (Bioss Antibodies, Inc., 1:200) at 4 °C overnight. On the next day, the sections were incubated with secondary antibody at room temperature for 1 h. The expression of Ki-67 was detected using a DAB immunohistochemistry color development kit (Sangon Biotech, China) following the manufacturer’s instructions. Cell images were captured under a fluorescence microscope (IX-51; Olympus) at 200x magnification.

### Statistical analysis

All data were presented as the mean ± standard deviation (SD). Each experiment was replicated for 3 times. Statistical analyses were performed using SPSS v. 19.0. The difference between two groups was determined using student’s *t*-test. Differences among multiple groups were determined using one-way analysis of variance (ANOVA). Spearman’s correlation analysis was used to analyze correlation in a data set. Kaplan-Meier curve was used to evaluate the overall survival of MM patients with different expression levels of BDNF-AS using the median expression level as the cutoff value (without considering the stage and prognosis of MM patients). *P* < 0.05 was considered as the significant threshold.

## Results

### BDNF-AS was upregulated, and miR-125a/b-5p were downregulated in MM serum and cell lines

**Fig. 1:**
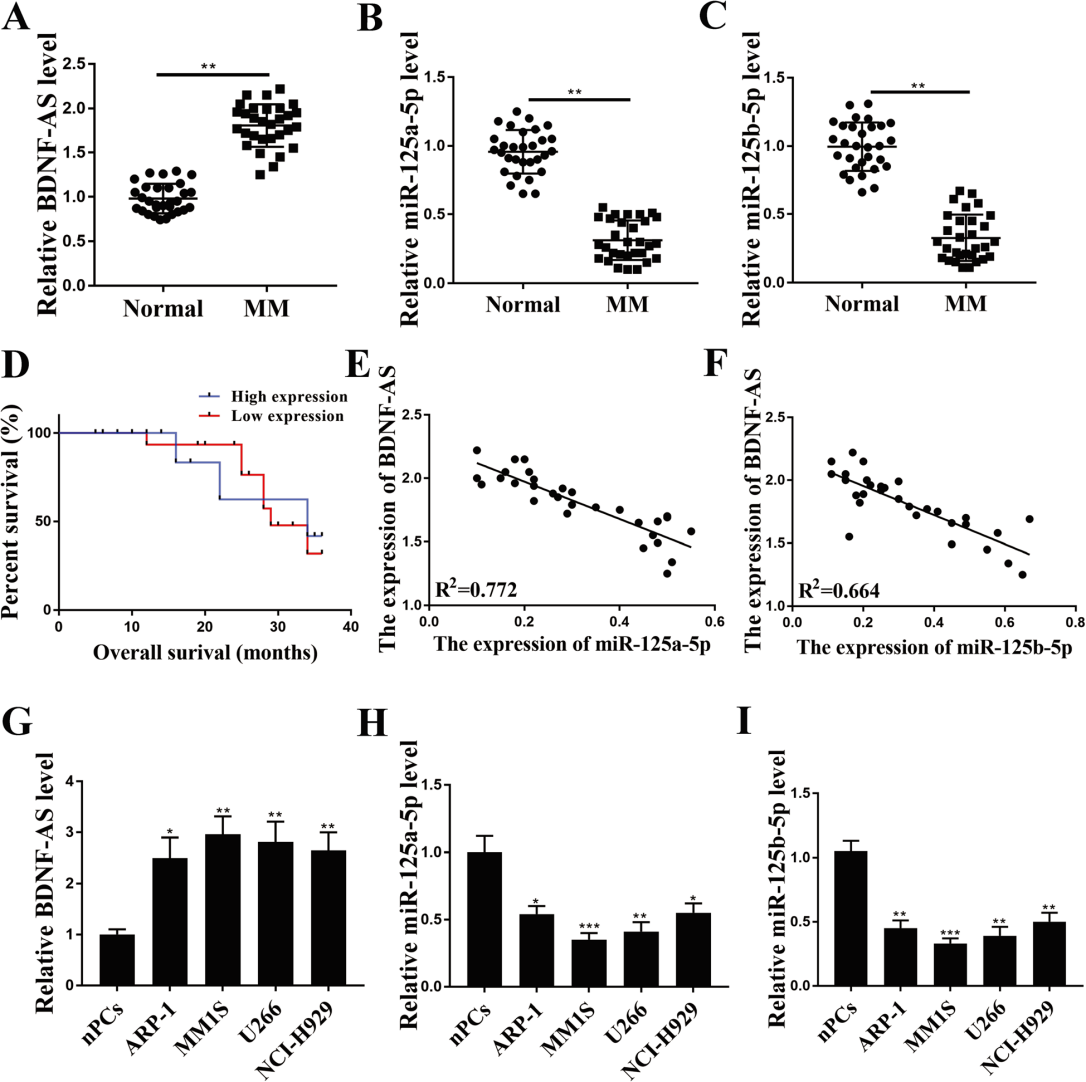
BDNF-AS was upregulated, and miR-125a/b-5p were downregulated in MM serum and cell lines. **a**-**c** The expression of BDNF-AS (**a**), miR-125a-5p (**b**) and miR-125b-5p (**c**) in MM serum and healthy controls were detected by qRT-PCR (*n* = 30). (**d**) The overall survival of MM patients was evaluated by Kaplan-Meier curve based on the expression of BDNF-AS. (**e** and **f**) The correlations between the expression of BDNF-AS and miR-125a-5p (**e**) as well as BDNF-AS and miR-125b-5p (**F**) in MM serum was determined by Spearman’s correlation analysis (*n* = 30). (**g**-**i**) The expression of BDNF-AS (**g**), miR-125a-5p (**h**) and miR-125b-5p (I) in MM cell lines and normal plasma cells (nPC) was detected by qRT-PCR. *p < 0.05, **p < 0.01, ***p < 0.001

To explore the role of BDNF-AS and miR-125a/b-5p in MM, we firstly detected their expression in the serum from MM patients (MM serum). The results showed that BDNF-AS was significantly upregulated in MM serum (*n* = 30) compared with that from healthy subjects (n = 30) (*p* < 0.01, Fig. 1A). However, the expression levels of BDNF-AS were significantly lower in the serum of MM patients at Stage III than that in Stage I and II (*p* < 0.01, Supplementary Table 1). Meanwhile, the expression of miR-125a-5p and miR-125b-5p were markedly downregulated in MM serum (n = 30) compared with that from healthy subjects (n = 30) (*p* < 0.01, Fig. 1B and C). All patients were divided into high and low BDNF-AS expression groups with the median expression level of BDNF-AS as the cutoff value. The survival analysis showed that high expression levels of BDNF-AS predicted a poorer overall survival of MM patients than that with low expression levels of BDNF-AS (*p* < 0.01, Fig. 1D). Spearman’s correlation analysis showed a significantly negative correlation between the expression of BDNF-AS and miR-125a/b-5p in MM tissues (*n* = 30, R^2^ = 0.772, Fig. 1E; R^2^ = 0.664, Fig. 1F). In addition, their expression levels in MM cell lines were also detected. The results showed that BDNF-AS was significantly upregulated (*p* < 0.05), while the expression of miR-125a-5p and miR-125b-5p were markedly downregulated in the 4 MM cell lines (*p* < 0.05, Fig. 1G-I). Due to the highest expression level of BDNF-AS and the lowest expression level of miR-125a/b-5p in MM.1S and U266 cells, these two cell lines were selected for the subsequent experiments.

### BDNF-AS acted as the sponge for miR-125a/b-5p

**Fig. 2:**
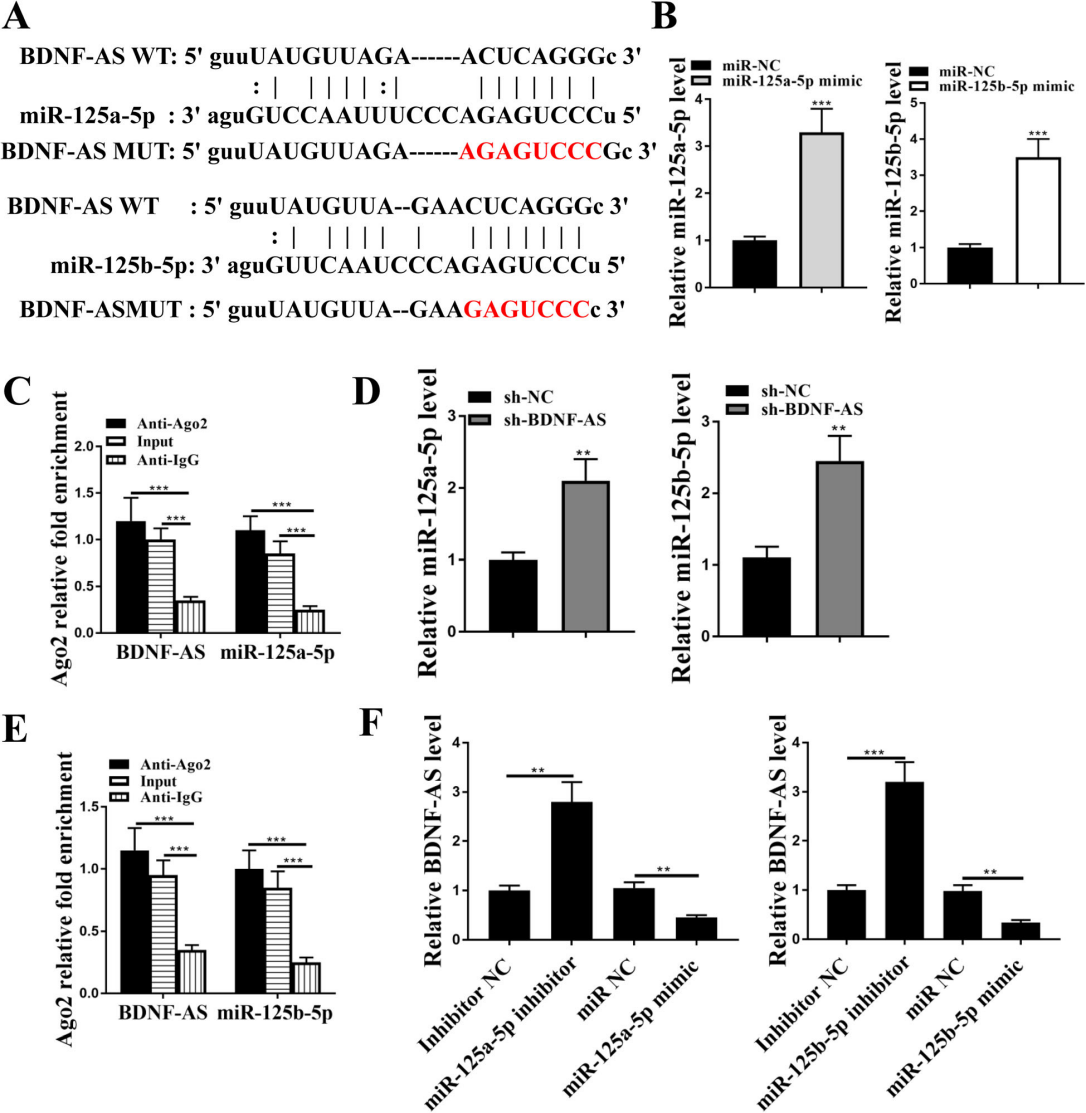
BDNF-AS acted as a sponge for miR-125a/b-5p. **a** The putative binding site between BDNF-AS and miR-125a/b-5p was predicted by Starbase 3.0. **b** HEK-293 T cells were transfected with miR-125a/b-5p mimics or inhibitors. The expression of miR-125a-5p and miR-125b-5p was detected by qRT-PCR. **c** The relative enrichment of BDNF-AS in HEK-293 T cells was determined by RIP assay. **d** The relative luciferase activity of BDNF-AS-WT or BDNF-AS-MUT in HEK-293 T cells was detected by dual luciferase reporter system. **e** HEK-293 T cells were transfected with the miR-125a/b-5p mimics or inhibitors. The expression of BDNF-AS was detected by qRT-PCR. **f** HEK-293 T cells were transfected with BDNF-AS shRNA or overexpression vector, and the expression of miR-125a/b-5p was detected by qRT-PCR. **p < 0.01, ***p < 0.001. ns: not significant

Starbase 3.0 was used to determine the binding relationship between BDNF-AS and miR-125a/b-5p. The prediction revealed a putative binding site between BDNF-AS and miR-125a-5p, as well as miR-125b-5p (Fig. 2A). To further confirm their relationship, miR-125a/b-5p mimics and inhibitors were transfected into HEK-293 T cells. qRT-PCR assay showed that the expression levels of miR-125a-5p and miR-125b-5p were significantly increased by mimics, and their expression levels were reduced by inhibitors (*p* < 0.001, Fig. 2B). The results of RIP assay showed that BDNF-AS was significantly enriched in cells transfected with miR-125a/b-5p mimics compared with that in cells transfected with miR-NC (*p* < 0.01, Fig. 2C), suggesting that BDNF-AS and miR-125a/b-5p were binding to Ago2. Then luciferase reporter assay was performed in HEK-293 T cells, and the results showed that overexpression of miR-125a-5p and miR-125b-5p both obviously reduced the relative luciferase activity of BDNF-AS-WT (*p* < 0.01, Fig. 2D), suggesting that BDNF-AS acted as the sponge of miR-125a/b-5p. In addition, overexpression or knockdown of miR-125a/b-5p did not affect the expression of BDNF-AS (Fig. 2E). And overexpression or knockdown of BDNF-AS had no effect on the expression of miR-125a/b-5p (Fig. 2F). Moreover, as shown in Supplementary Table 2, the copy numbers of BDNF-AS and miR-125a/b-5p per cell were comparable with their stoichiometric interaction. These results demonstrated that BDNF-AS served as a sponge for miR-125a/b-5p.

### Knockdown of BDNF-AS significantly reduced proliferation and induced apoptosis in MM cells in vitro

**Fig. 3:**
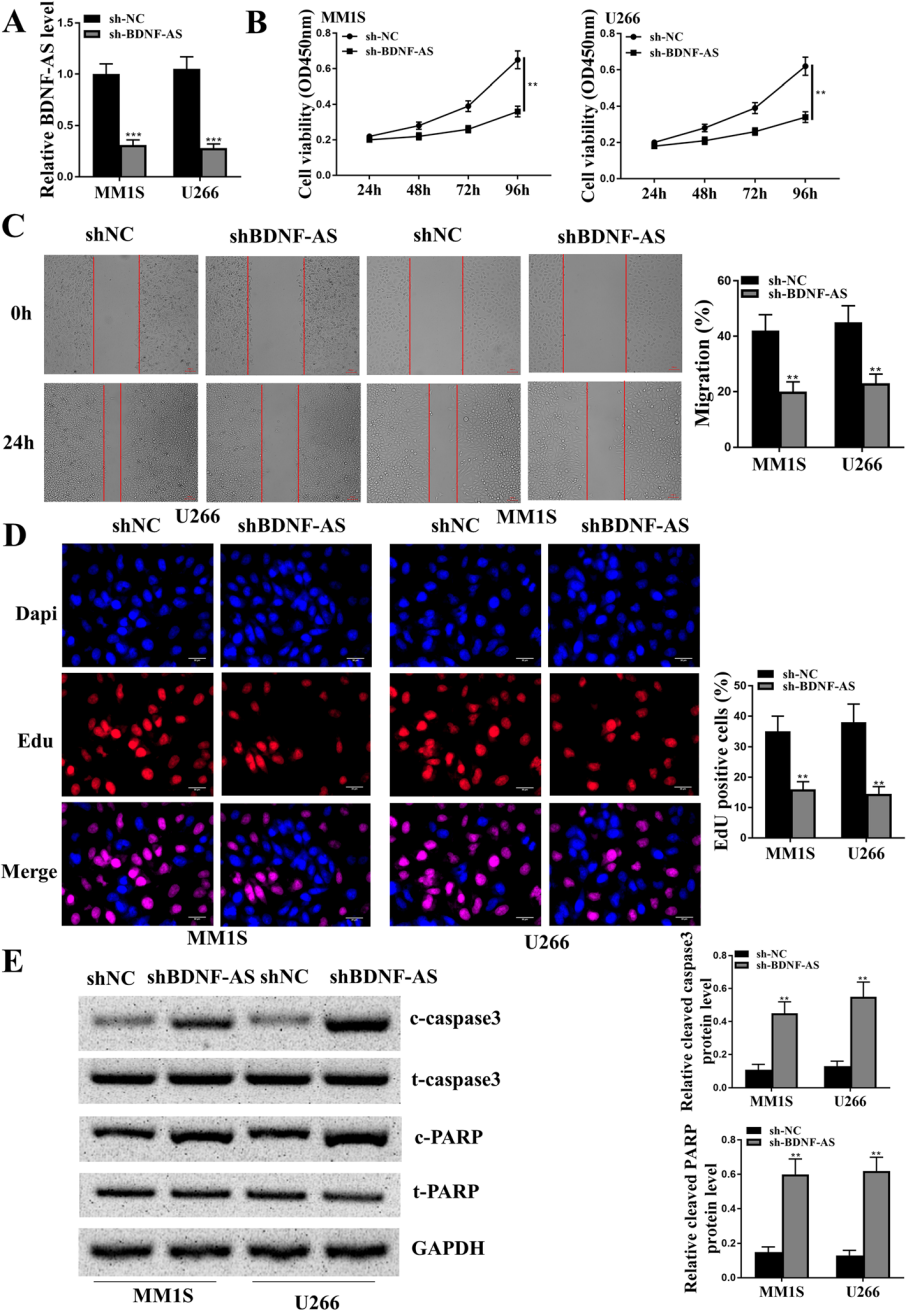
Knockdown of BDNF-AS significantly reduced proliferation of MM cells. MM.1S and U266 cells were transfected with the lentiviral vector carrying sh-BDNF-AS and sh-NC. **a** The expression of BDNF-AS was detected by qRT-PCR. **b** Cell viability was evaluated by CCK-8 assay. **c** Cell migration ability was evaluated by wound healing assay. Scale bar = 100 μm. **d** Cell proliferation was evaluated by EdU staining assay. Scale bar = 30 μm. **e** The expression of apoptosis-related proteins was evaluated by Western blot. Relative cleaved caspase-3 means cleaved caspase-3/total caspase-3, and relative cleaved PARP means cleaved PARP/total PARP. **p < 0.01, ***p < 0.001

To explore the role of BDNF-AS in MM, MM.1S and U266 cells were transfected with the lentiviral vector carrying sh-BDNF-AS and sh-NC. The transfection efficiency was evaluated by qRT-PCR, and the results showed that sh-BDNF-AS significantly reduced the expression levels of BDNF-AS compared with sh-NC in MM.1S (*p* < 0.001) and U266 cells (*p* < 0.001, Fig. 3A). By performing CCK-8 assay, we found that knockdown of BDNF-AS significantly decreased the viability of MM.1S (*p* < 0.01) and U266 cells compared with sh-NC (*p* < 0.01, Fig. 3B). The wound healing assay results also showed that knockdown of BDNF-AS significantly reduced the migration capacity of both MM.1S and U266 cells compared with sh-NC (*p* < 0.01, Fig. 3C). The EdU staining assay showed that knockdown of BDNF-AS markedly reduced the percentage of EdU positive cells in both MM.1S (*p* < 0.01) and U266 cells compared with sh-NC (*p* < 0.01, Fig. 3D). Furthermore, the expression levels of apoptosis-related proteins were evaluated using Western blot analysis, and the results showed that knockdown of BDNF-AS significantly increased the ratio of cleaved caspase 3/total caspase 3 and cleaved PARP/total PARP in both MM.1S and U266 cells (all *p* < 0.01) (Fig. 3E). These results demonstrated that knockdown of BDNF-AS significantly reduced proliferation and promoted apoptosis of MM cells.

### Knockdown of miR-125a-5p reversed the effects of sh-BDNF-AS on proliferation and apoptosis of MM cells

**Fig. 4:**
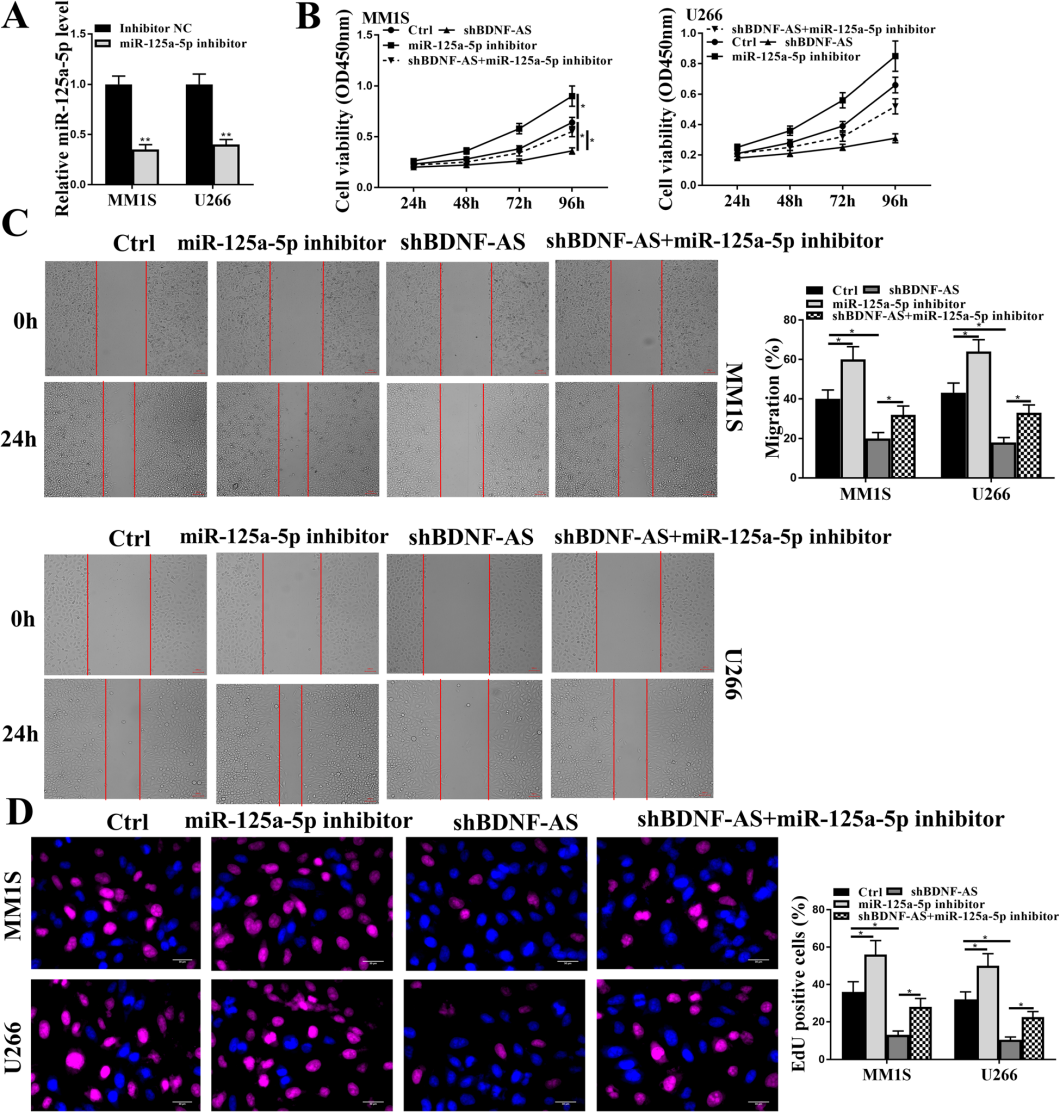
Knockdown of miR-125a-5p attenuated sh-BDNF-AS inhibited MM cell proliferation. **a** MM.1S and U266 cells were transfected with miR-125a-5p inhibitor or inhibitor NC, and the transfection efficiency was evaluated by qRT-PCR. **b**-**d** MM.1S and U266 cells were transfected sh-BDNF-AS, miR-125a-5p inhibitor, or co-transfected with sh-BDNF-AS and miR-125a-5p inhibitor. **b** Cell viability was evaluated by CCK-8 assay. **c** Cell migration ability was evaluated by wound healing assay. Scale bar = 100 μm. **d** Cell proliferation was evaluated by EdU staining assay. Scale bar = 30 μm. *p < 0.05, **p < 0.01

To further determine BDNF-AS/miR-125a-5p-mediated effects on MM cells, MM.1S and U266 cells were transfected with miR-125a-5p inhibitor or inhibitor NC, and the transfection efficiency was evaluated by qRT-PCR. The results showed that miR-125a-5p inhibitor significantly reduced the expression levels of miR-125a-5p compared with inhibitor NC in MM.1S and U266 cells (*p* < 0.01, Fig. 4A). Then MM.1S and U266 cells were transfected with sh-BDNF-AS, miR-125a-5p inhibitor, or co-transfected with sh-BDNF-AS and miR-125a-5p inhibitor. CCK-8 assay showed that knockdown of BDNF-AS reduced the viability of MM.1S and U266 cells (*p* < 0.05), miR-125a-5p inhibitor obviously enhanced their viability (*p* < 0.05), and co-transfection of sh-BDNF-AS and miR-125a-5p inhibitor significantly reversed the inhibitory effects of sh-BDNF-AS on the viability of MM.1S and U266 cells (*p* < 0.05, Fig. 4B). In addition, knockdown of BDNF-AS significantly reduced the migration rate of MM.1S and U266 cells (*p* < 0.05), miR-125a-5p inhibitor significantly increased their migration rate (*p* < 0.05), and co-transfection of sh-BDNF-AS and miR-125a-5p inhibitor significantly reversed the inhibitory effect of sh-BDNF-AS on the migration rate of MM.1S and U266 cells (*p* < 0.05, Fig. 4C). Moreover, EdU staining assay showed that the number of EdU positive MM.1S and U266 cells was significantly reduced by knockdown of BDNF-AS (*p* < 0.05) and increased by miR-125a-5p inhibitor (*p* < 0.05). And co-transfection of sh-BDNF-AS and miR-125a-5p inhibitor obviously attenuated the inhibitory effect of sh-BDNF-AS on the number of EdU positive cells (*p* < 0.05, Fig. 4D). Furthermore, the expression levels of apoptosis-related proteins were evaluated by Western blot, and the results showed that miR-125a-5p inhibitor significantly reduced the ratio of cleaved caspase 3/total caspase 3 and cleaved PARP/total PARP (all *p* < 0.05), and co-transfection of sh-BDNF-AS and miR-125a-5p inhibitor obviously reversed the effects of sh-BDNF-AS on the expression of apoptosis-related proteins (all *p* < 0.05) but not that of total caspase 3 and total PARP in MM.1S and U266 cells (Supplementary Fig. 1A). These results revealed that downregulation of miR-125a-5p reversed the effects of knockdown of BDNF-AS on the proliferation and apoptosis of MM cells in vitro.

### Knockdown of miR-125b-5p reversed the effects of sh-BDNF-AS on proliferation and apoptosis of MM cells

**Fig. 5:**
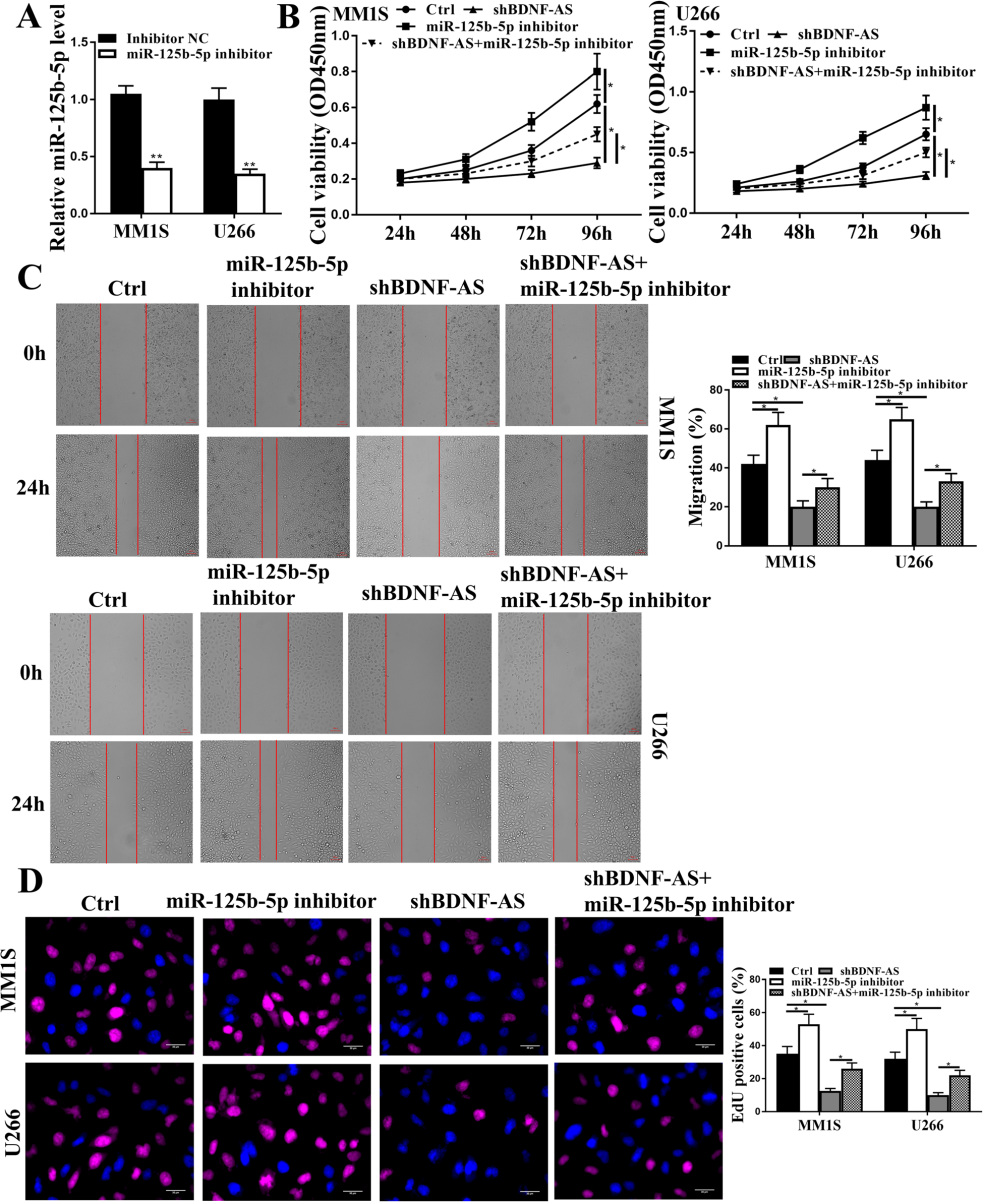
Knockdown of miR-125b-5p attenuated sh-BDNF-AS inhibited MM cell proliferation. **a** MM.1S and U266 cells were transfected with miR-125b-5p inhibitor or inhibitor NC, and the transfection efficiency was evaluated by qRT-PCR. **b**-**d** MM.1S and U266 cells were transfected sh-BDNF-AS, miR-125b-5p inhibitor, or co-transfected with sh-BDNF-AS and miR-125b-5p inhibitor. **b** Cell viability was evaluated by CCK-8 assay. **c** Cell migration ability was evaluated by wound healing assay. Scale bar = 100 μm. **d** Cell proliferation was evaluated by EdU staining assay. Scale bar = 30 μm. *p < 0.05, **p < 0.01

Similarly, we also explored whether the effect of BDNF-AS was mediated by miR-125b-5p. MiR-125b-5p inhibitor or inhibitor NC was transfected into MM.1S and U266 cells, and qRT-PCR assay showed that miR-125b-5p inhibitor significantly reduced the expression levels of miR-125b-5p compared with inhibitor NC in MM.1S and U266 cells (*p* < 0.01, Fig. 5A). Then MM.1S and U266 cells were transfected with sh-BDNF-AS, miR-125b-5p inhibitor, or co-transfected with sh-BDNF-AS and miR-125b-5p inhibitor. CCK-8 assay showed that the viability of MM.1S and U266 cells were significantly reduced by knockdown of BDNF-AS (*p* < 0.05) and enhanced by miR-125b-5p inhibitor (*p* < 0.05). Co-transfection of sh-BDNF-AS and miR-125b-5p inhibitor significantly reversed the inhibitory effect of sh-BDNF-AS on cell viability (*p* < 0.05, Fig. 5B). Wound healing assay showed that knockdown of BDNF-AS significantly reduced cell migration rate (*p* < 0.05), miR-125b-5p inhibitor significantly increased cell migration rate compared with that in the control group (*p* < 0.05), and co-transfection of sh-BDNF-AS and miR-125b-5p inhibitor reversed the inhibitory effect of sh-BDNF-AS on cell migration (*p* < 0.05, Fig. 5C). Meanwhile, the number of EdU positive cells was significantly reduced by knockdown of BDNF-AS (*p* < 0.05) and increased by miR-125b-5p inhibitor (*p* < 0.05). Co-transfection of sh-BDNF-AS and miR-125b-5p inhibitor obviously attenuated the inhibitory effect of sh-BDNF-AS on the number of EdU positive cells (*p* < 0.05, Fig. 5D). In addition, the expression levels of apoptosis-related proteins were evaluated by Western blot analysis. The results showed that miR-125b-5p inhibitor significantly reduced the ratio of cleaved caspase 3/total caspase 3 and cleaved PARP/total PARP (all *p* < 0.01), and co-transfection of sh-BDNF-AS and miR-125a-5p inhibitor obviously reversed the effects of sh-BDNF-AS on the expression of apoptosis-related proteins (all *p* < 0.01) but exhibited no effects on total caspase 3 and total PARP in MM.1S and U266 cells (Supplementary Fig. 1B). These results demonstrated that downregulation of miR-125b-5p reversed the effects of knockdown of BDNF-AS on proliferation and apoptosis of MM cells in vitro.

### Bcl-2 was the target of miR-125a-5p and miR-125b-5p

**Fig. 6:**
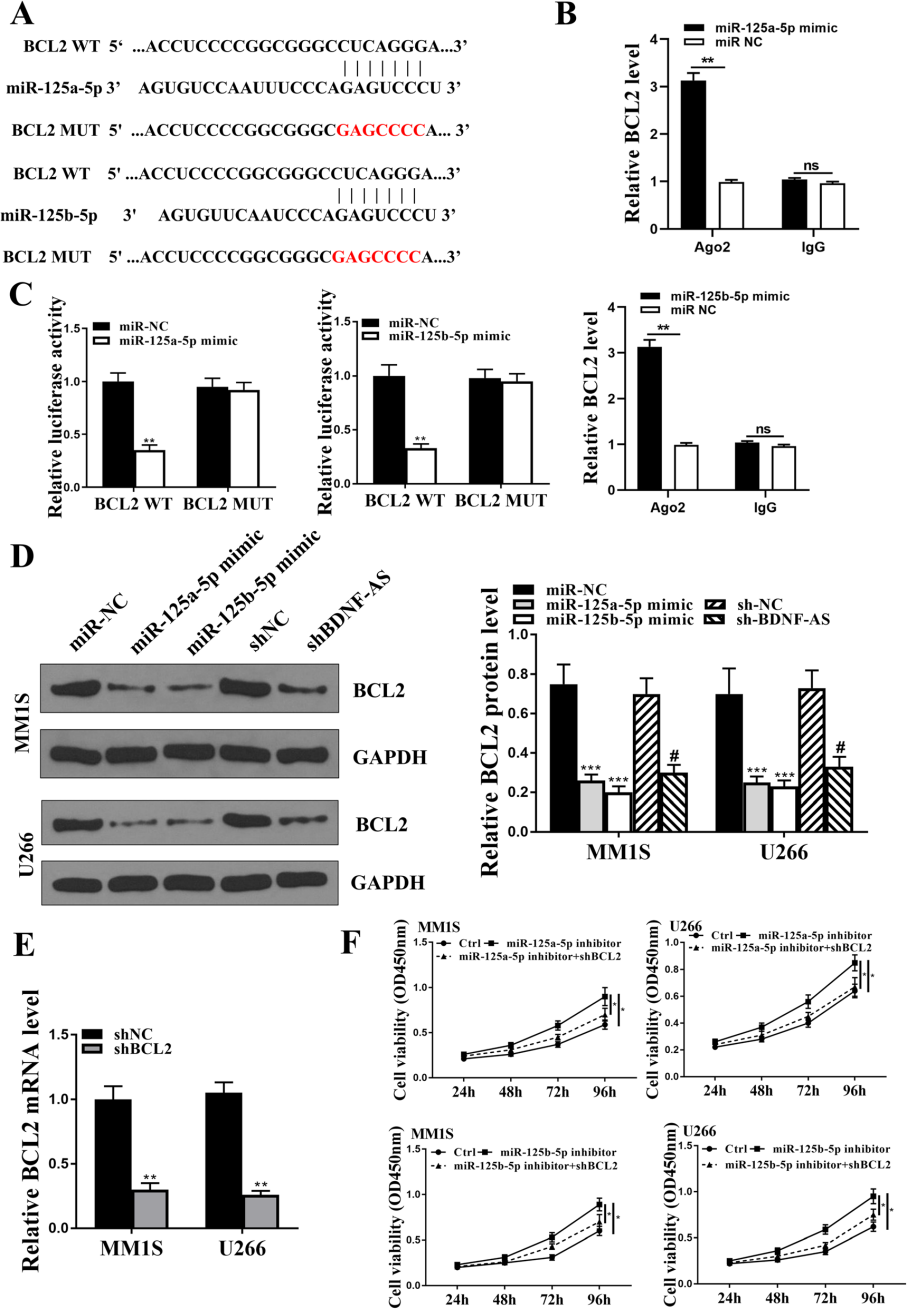
Bcl-2 was a target of miR-125a-5p and miR-125b-5p. **a** The putative binding site between miR-125a/b-5p and Bcl-2 was predicted by Starbase 3.0. **b** The relative luciferase activity of Bcl-2-WT or Bcl-2-MUT in HEK-293 T cells was detected by dual luciferase reporter system. **c** The enrichment of BCL-2 in HEK-293 T cells was detected by qRT-PCR. **d** The expression of Bcl-2 in MM.1S and U266 cells was detected by Western blot. **e** MM.1S and U266 cells were transfected with lentiviral vectors carrying sh-Bcl-2 and sh-NC, and the transfection efficiency was detected by qRT-PCR. **f** MM.1S and U266 cells were transfected miR-125a-5p inhibitor, miR-125b-5p inhibitor, or co-transfected with miR-125a-5p inhibitor and sh-Bcl-2, or co-transfected with miR-125b-5p inhibitor and sh-Bcl-2. Cell viability was evaluated by CCK-8 assay. *p < 0.05, **p < 0.01, ***p < 0.001 vs miR-NC group/miR-125a/b-5p inhibitor group; #*p* < 0.05 vs sh-NC group. ns: not significant

**Fig. 7:**
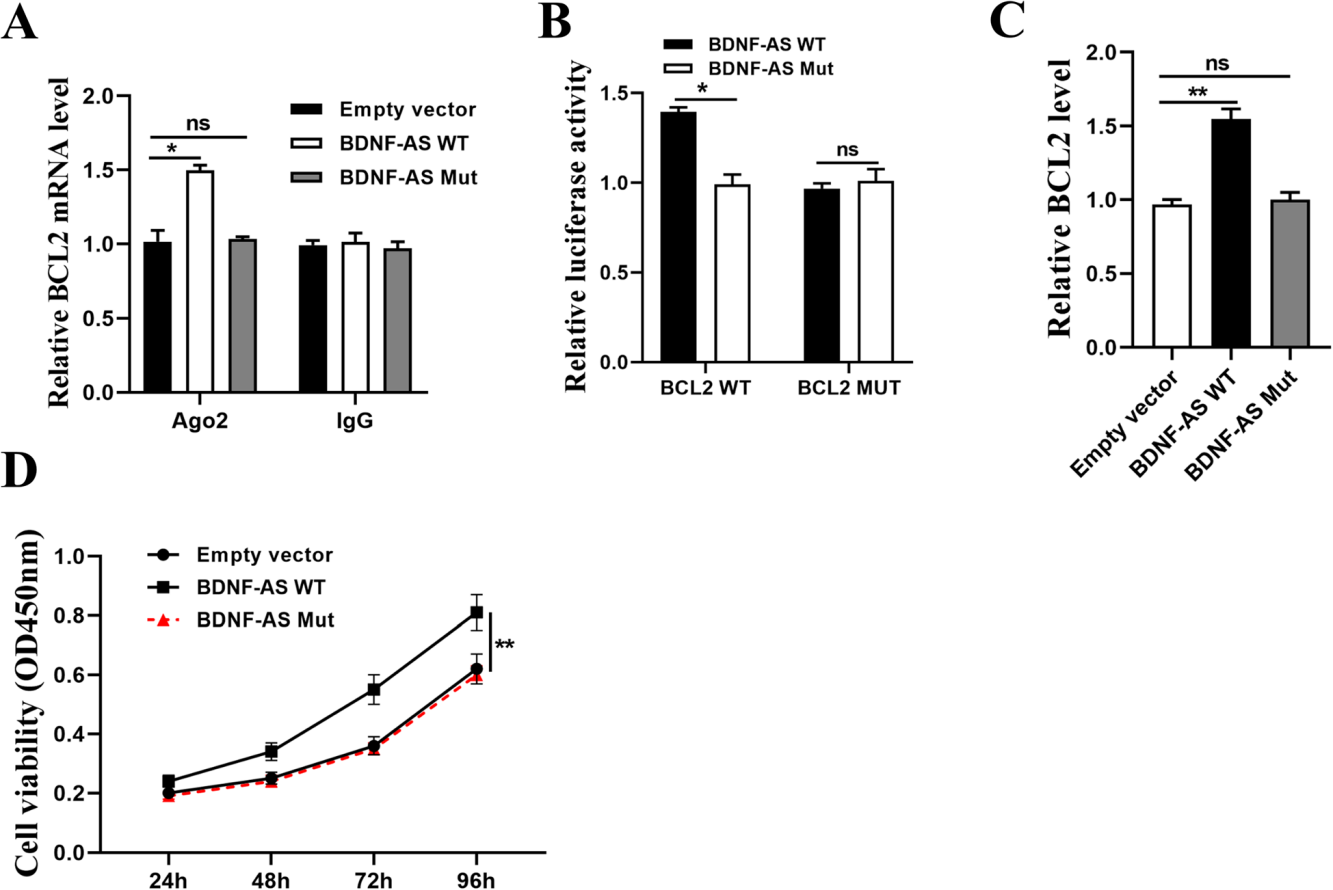
BDNF-AS increased the expression levels of BCL-2 by sponging miR-125a/b-5p. Cells were transfected with BDNF-AS WT and BDNF-AS Mut. **a** The enrichment of BCL-2 was detected by RT-qPCR in Ago2 RIP assay. **b** The luciferase activity was detected by dual luciferase reporter system. **c** The expression of BCL-2 mRNA was detected by RT-qPCR. **d** The cell viability was detected by CCK-8 assay. *p < 0.05 and **p < 0.01 vs empty vector; ns: not significant

To further investigate the specific mechanism of BDNF-AS in MM, Starbase 3.0 was used to predict the potential targets of miR-125a/b-5p. It showed that Bcl-2 had a putative binding site with miR-125a-5p and miR-125b-5p (Fig. 6A). In addition, Ago2 RIP assay was performed, and the results showed that BCL2 was significantly enriched in the cells transfected with miR-125a/b-5p mimics compared with that in cells transfected with miR-NC (*p* < 0.01, Fig. 6B). Luciferase reporter assay was also performed in HEK-293 T cells, and the results showed that overexpression of miR-125a-5p or miR-125b-5p decreased the relative luciferase activity of Bcl-2-WT compared with miR-NC (*p* < 0,01) but had no effect on Bcl-2-MUT (Fig. 6C). Western blot analysis showed that both miR-125a-5p and miR-125b-5p mimics significantly decreased the expression levels of Bcl-2 compared with miR-NC in MM.1S and U266 cells (*p* < 0.001), and knockdown of BDNF-AS also decreased the expression levels of Bcl-2 compared with sh-NC in these two cells (*p* < 0.05, Fig. 6D). Next, Bcl-2 was silenced in MM.1S and U266 cells by the transfection of lentiviral vector carrying sh-Bcl-2 and sh-NC, and qRT-PCR assay showed that sh-Bcl-2 significantly reduced the expression levels of Bcl-2 in MM.1S and U266 cells compared with sh-NC (*p* < 0.01, Fig. 6E). To determine whether the effect of miR-125a/b-5p in MM was mediated by Bcl-2, MM.1S and U266 cells were transfected with miR-125a-5p inhibitor, miR-125b-5p inhibitor, or co-transfected with miR-125a-5p inhibitor and sh-Bcl-2, or co-transfected with miR-125b-5p inhibitor and sh-Bcl-2. CCK-8 assay showed that miR-125a-5p inhibitor and miR-125b-5p inhibitor significantly enhanced cell viability compared with that in the control (*p* < 0.05), and this effect was obviously blunted by co-transfection of miR-125b-5p inhibitor and sh-Bcl-2, or miR-125b-5p inhibitor and sh-Bcl-2 (*p* < 0.05, Fig. 6F). These results demonstrated that Bcl-2 was the target of miR-125a/b-5p. To confirm the relationship among BDNF-AS, miR-125a/b-5p, and BCL-2, further experiments were performed. NM cells were transfected with the wild-type (WT) or mutant (Mut) BDNF-AS overexpression vector. Ago2 RIP assay showed that overexpression of BDNF-AS-WT increased the enrichment of BCL-2 binding to Ago2, while overexpression of BDNF-AS-Mut had no effect on the enrichment of BCL-2 (Fig. 7A). In addition, NM cells were transfected with luciferase reporters containing BCL2 3′-UTRs and the wild-type (WT) or mutant (Mut) BDNF-AS overexpression vector. The results showed that overexpression of BDNF-AS-WT increased the luciferase activity in cells co-transfected with BCL2 3′-UTRs, while overexpression of BDNF-AS-Mut did not affect the luciferase activity of cells co-transfected with BCL2 3′-UTRs (Fig. 7B). Moreover, overexpression of BDNF-AS-WT increased the expression levels of BCL2 and enhanced cancer cell viability, while overexpression of BDNF-AS-WT had no effect on the expression of BCL2 and cell viability (Fig. 7C-D). Taken together, these data suggested that BDNF-AS functions in NM cells by regulating the miR-125a/b-5p/BCL2 axis.

### Knockdown of BDNF-AS suppressed tumor growth in vivo

**Fig. 8:**
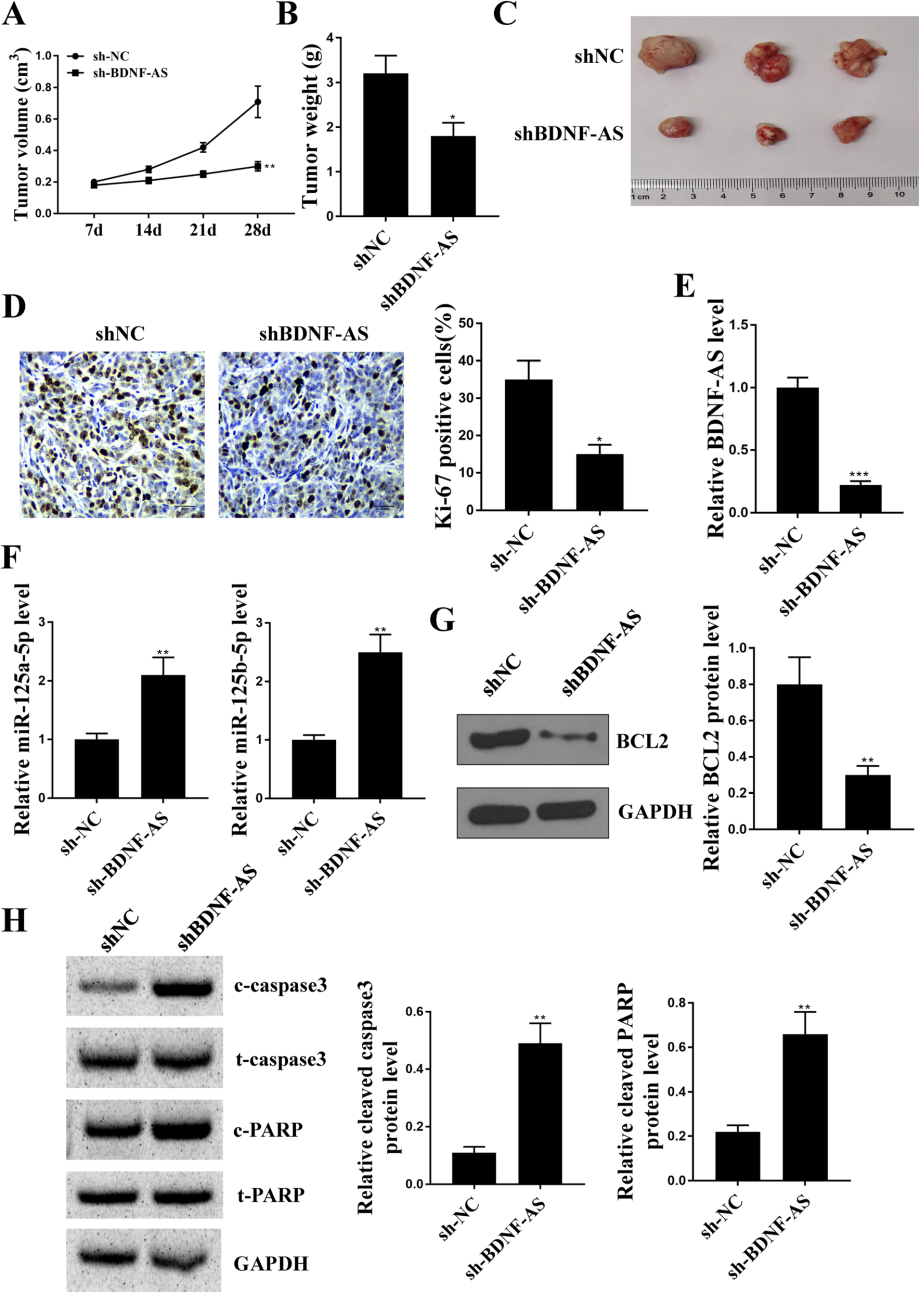
Knockdown of BDNF-AS suppressed tumor growth in vivo. **a** Tumor volume was evaluated every week for 4 weeks. **b** Tumor weight was evaluated after 4 weeks. **c** Representative images of xenograft tumors. **d** The expression of Ki-67 in tumor tissues was evaluated by IHC staining. Scale bar = 30 μm. **e** and **f** The expression of BDNF-AS (**e**) and miR-125a/b-5p (**f**) in tumor tissues was detected by qRT-PCR. (**g**) The expression of Bcl-2 in tumor tissues was detected by Western blot. (H) The levels of cleaved caspase 3 (c-caspase 3), total caspase 3 (t-caspase 3), cleaved PARP (c-PARP) and total PARP (t-PARP) in tumor tissues was evaluated by Western blot. Four mice in each group. Relative cleaved caspase-3 means cleaved caspase-3/total caspase-3, and relative cleaved PARP means cleaved PARP/total PARP. *p < 0.05, **p < 0.01, ***p < 0.001

To further determine the protective role of knockdown of BDNF-AS in MM in vivo, MM.1S cells stably transfected with sh-BDNF-AS or sh-NC were subcutaneously injected into nude mice to establish the xenograft tumor model. We found that knockdown of BDNF-AS effectively reduced tumor volume (*p* < 0.01) and tumor weight compared with that in sh-NC group (*p* < 0.05, Fig. 8A-C). Meanwhile, IHC staining in the tumors revealed that the number of Ki-67-positive cells was obviously reduced in sh-BDNF-AS group compared with that in sh-NC group (*p* < 0.05, Fig. 8D). The expression of BDNF-AS, miR-125a-5p, and miR-125b-5p in tumor tissues were detected by qRT-PCR, and the results showed that BDNF-AS was significantly downregulated (*p* < 0.001), while miR-125a-5p and miR-125b-5p in tumor tissues were markedly upregulated in sh-BDNF-AS injected mice compared with that in sh-NC injected mice (*p* < 0.01, Fig. 8E and F). Moreover, the expression of Bcl-2 protein in tumor tissues was significantly downregulated in sh-BDNF-AS injected mice compared with that in sh-NC injected mice (*p* < 0.01, Fig. 8G). In addition, the expression of apoptosis-related protein in tumor tissues were evaluated by Western blot analysis, and the results showed that knockdown of BDNF-AS significantly increased the ratio of cleaved caspase 3/total caspase 3 and cleaved PARP/total PARP (both *p* < 0.01) (Fig. 8H). These results demonstrated that knockdown of BDNF-AS suppressed tumor growth in vivo.

## Discussion

Recently, increasing attention has been paid to MM due to its severe health challenges [42]. Studies have revealed the dysregulation of ncRNAs in MM and their potentials as therapeutic targets [43]. In this study, we found that BDNF-AS was significantly upregulated in MM serum, and knockdown of BDNF-AS effectively inhibited proliferation and induced apoptosis of MM cells in vitro and suppressed tumor growth in vivo. Specifically, knockdown of BDNF-AS lead to reduced expression levels of Bcl2 protein and decreased rate of cell apoptosis and proliferation in a miR-125-5p dependent manner. Importantly, our study revealed a new function of BDNF-AS in MM, which extended our understanding of the pathology of the development of MM.

Extensive studies have revealed that lncRNAs have typical molecular functions as competitive endogenous RNAs (ceRNAs) or miRNA sponges to participate in cancer development [44]. BDNF-AS is a well-understood lncRNA associated with the progression of various human diseases. For example, BDNF-AS is significantly downregulated in esophageal cancer tissues, and overexpression of BDNF-AS effectively inhibits proliferation, migration, invasion, and epithelial-to-mesenchymal transition (EMT) of esophageal cancer cells by targeting miR-214 [45]. In addition, BDNF-AS is markedly downregulated in human prostate cancer tissues, and low expression levels of BDNF-AS is correlated with the poor prognosis and shorter overall survival of prostate cancer patients [46]. These findings suggested that BDNF-AS might be involved in the progression of various cancers. However, the functions of BDNF-AS in MM have not been reported. In this study, we aimed to investigate the role of BDNF-AS in MM and found that BDNF-AS was markedly upregulated in MM. Moreover, knockdown of BDNF-AS effectively inhibited the progression of MM both in vitro and in vivo, indicating that BDNF-AS might be a therapeutic target for MM.

It has been reported that lncRNAs often compete with miRNAs response elements to inhibit their functions and activities of miRNAs and then silence miRNAs at the post-transcriptional level, followed by the regulation of downstream mRNAs [47]. It was reported that BDNF-AS could promote autophagy and apoptosis in Parkinson’s disease by directly regulating miR-125b-5p [27], revealing that miR-125b-5p was a target of BDNF-AS. MiR-125a-5p and miR-125b-5p are identified as the two members of the miRNA-125 family, and their aberrant expression was tightly associated with tumorigenesis and tumor development, including MM [48]. Significant downregulation of miR-125b-5p was observed in MM, and a low expression level of miR-125b-5p was associated with apoptotic and autophagy-related cell death in MM in vitro [49]. Meanwhile, downregulation of miR-125a-5p effectively inhibits proliferation and migration of MM cells in vitro [24]. To determine whether BDNF-AS participated in MM development by regulating miR-125a-5p or miR-125b-5p. We used Starbase to predict the putative binding site between BDNF-AS and miR-125a/b-5p. A putative binding site between miR-125a/b-5p and BDNF-AS was predicted, indicating that miR-125a/b-5p might mediate the function of BDNF-AS in MM. Luciferase reporter assay and RIP assay further confirmed their binding relationship. Moreover, knockdown of miR-125a/b-5p attenuated the inhibitory effects of knockdown of BDNF-AS on proliferation and apoptosis of MM cells. These results confirmed the role of BDNF-AS/miR-125a/b-5p in MM.

Anti-apoptotic protein Bcl-2 has a protective role in tumor cells by promoting tumor cell growth and proliferation and inhibiting apoptosis [32]. As a key element in cancer development, the miRNA/Bcl-2 signaling has been widely identified in different types of human disease, such as miR-34a in age-related hearing loss [50], miR-21 in gastrointestinal stromal tumor [51], miR-181b-5p in glioma [52] and miR-497 in MM [53]. A previous study demonstrated that miR-125a-5p inhibited cell proliferation and induced apoptosis in hepatocellular carcinoma by targeting Bcl-2-like-2 protein [54]. The prediction by Starbase indicated that Bcl-2 might be a target of miR-125a/b-5p. Therefore, we speculated that miR-125a/b-5p affected MM development by targeting Bcl-2. Our results revealed that miR-125a/b-5p inhibitor significantly enhanced proliferation of MM.1S and U266 cells, while these effects were obviously reversed by knockdown of Bcl-2. These results demonstrated that the effects of BDNF-AS/miR-125a/b-5p in MM were partially mediated by Bcl-2.

Bcl-2 is closely associated with invasion and migration of tumor cells. Knockdown of endogenous Bcl-2 significantly reduces hypoxia-induced cell invasion and migration in human osteosarcoma [55]. Overexpression of Bcl-2 attenuates the inhibitory effects of overexpression of miR-15 on cell invasion and migration in thyroid cancer [34]. However, whether Bcl-2 mediates the effects of BDNF-AS on MM cell invasion and migration needs to be further explored in the future.

However, our study has some limitations. The sample size of MM patients is small, which can impair the credibility of our findings in the present study. In addition, our study found that MM patients had high expression levels of BDNF-AS in serum sample. However, the expression levels of BDNF-AS were significantly lower in the serum of MM patients at Stage III than that in Stage I and II. Due to the limited sample size, the effect of the stage of cancer in the survival analysis was not investigated here. Our future study will collect more samples to further verify our findings and explore the effect of the cancer stage on the function of BDNF-AS in the prognosis of MM.

## Conclusion

In summary, our study demonstrated that the BDNF-AS/miR-125a/b-5p/Bcl-2 axis was closely associated with the progression of MM, suggesting that this axis might be a potential diagnostic and therapeutic target for MM.

## Supplementary Information

**Additional file 1: Supplementary Table 1.** Difference in BDNF-AS expression in multiple myeloma patients grouped by clinicopathological characteristics.

**Additional file 2: Supplementary Table 2.** The copy numbers of BDNF-AS and miR-125a/b-5p in NM cells.

**Additional file 3: Supplementary Fig. 1.** MiR-125a/b-5p inhibitor significantly reversed the effects of BDNF-AS knockdown on apoptosis of MM cells. (A) MM.1S and U266 cells were transfected with sh-BDNF-AS, or co-transfected with sh-BDNF-AS and miR-125a-5p inhibitor. (B) MM.1S and U266 cells were transfected with sh-BDNF-AS, or co-transfected with sh-BDNF-AS and miR-125b-5p inhibitor. The levels of cleaved caspase 3 (c-caspase 3), total caspase 3 (t-caspase 3), cleaved PARP (c-PARP) and total PARP (t-PARP) was evaluated by Western blot. Relative cleaved caspase-3 means cleaved caspase-3/total caspase-3, and relative cleaved PARP means cleaved PARP/total PARP. *p < 0.05, **p < 0.01.

## Data Availability

The analyzed data sets generated during the present study are available from the corresponding author on reasonable request.
