# Supplementary material for: Knockdown of lncRNA BDNF-AS inhibited the progression of multiple myeloma by targeting the miR-125a/b-5p-BCL2 axis
Source: Immun Ageing. 2022 Jan 3;19:3. doi: 10.1186/s12979-021-00258-5 (PMC8722203; doi:10.1186/s12979-021-00258-5)
Supplement: Supplementary file 1 — Additional file 1: Supplementary Table 1. Difference in BDNF-AS expression in multiple myeloma patients grouped by clinicopathological characteristics. [file 12979_2021_258_MOESM1_ESM.docx]

**Supplementary** **Table 1** Difference in BDNF-AS expression in multiple myeloma patients grouped by clinicopathological characteristics

| **Clinicopathological characteristics** | **Number of patients** | **Relative BDNF-AS level in serum ^a^** | ***P*- value** |
| --- | --- | --- | --- |
| **Gender** | | | |
| Male | 14 | 0.69 ± 0.10 | 0.188 |
| Female | 16 | 0.48 ± 0.08 |  |
| **Age (year)** | | | |
| < 45 | 15 | 0.68 ± 0.09 | 0.706 |
| ≥ 45 | 15 | 0.72 ± 0.11 |  |
| **M protein** | | | |
| IgG | 7 | 0.61 ± 0.09 | 0.801 |
| IgA | 8 | 0.64 ± 0.12 |  |
| Light chain | 15 | 0.65 ± 0.08 |  |
| **ISS stage** | | | |
| I | 5 | 0.89 ± 0.18 | 0.01 |
| II | 15 | 0.95 ± 0.11 |  |
| III | 10 | 0.69 ± 0.07 |  |
| **Durie-Salmon stage** | | | |
| I | 7 | 1.12 ± 0.12 | 0.01 |
| II | 13 | 0.89 ± 0.08 |  |
| III | 10 | 0.62 ± 0.08 |  |
| **Anemia** | | | |
| With | 22 | 0.62 ± 0.09 | 0.261 |
| Without | 8 | 0.74 ± 0.13 |  |
| **Bone disease** | | | |
| With | 29 | 0.66 ± 0.08 | 0.52 |
| Without | 18 | 0.66 ± 0.08 |  |
| **Cytogenetic abnormality** | | | |
| With | 9 | 0.76 ± 0.11 | 0.322 |
| Without | 21 | 0.65 ± 0.09 |  |

^a^ The relative expression of BDNF-AS to GAPDH was calculated using 2^−∆∆Ct^ method and shown as mean ± SD between different clinicopathological characteristics in patients.
