# Supplementary material for: Knockdown of lncRNA BDNF-AS inhibited the progression of multiple myeloma by targeting the miR-125a/b-5p-BCL2 axis
Source: Immun Ageing. 2022 Jan 3;19:3. doi: 10.1186/s12979-021-00258-5 (PMC8722203; doi:10.1186/s12979-021-00258-5)
Supplement: Supplementary file 2 — Additional file 2: Supplementary Table 2. The copy numbers of BDNF-AS and miR-125a/b-5p in NM cells. [file 12979_2021_258_MOESM2_ESM.docx]

**Supplementary** **Table 2** The copy numbers of BDNF-AS and miR-125a/b-5p in NM cells.

| Cell line | Copy number per cell | | |
| --- | --- | --- | --- |
|  | BDNF-AS | miR-125a-5p | miR-125b-5p |
| MM1S | 46 | 33 | 35 |
| U266 | 43 | 31 | 37 |
