# Supplementary figures and images for: Knockdown of lncRNA BDNF-AS inhibited the progression of multiple myeloma by targeting the miR-125a/b-5p-BCL2 axis
Source: Immun Ageing. 2022 Jan 3;19:3. doi: 10.1186/s12979-021-00258-5 (PMC8722203; doi:10.1186/s12979-021-00258-5)

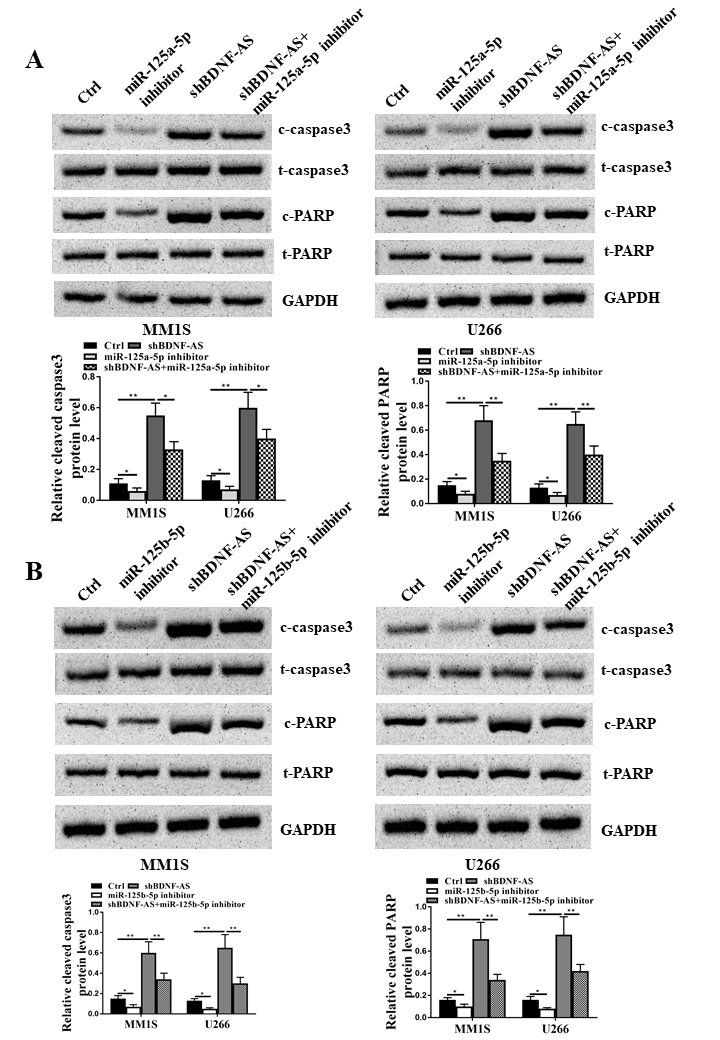

Supplement: Supplementary file 3 — Additional file 3: Supplementary Fig. 1. MiR-125a/b-5p inhibitor significantly reversed the effects of BDNF-AS knockdown on apoptosis of MM cells. (A) MM.1S and U266 cells were transfected with sh-BDNF-AS, or co-transfected with sh-BDNF-AS and miR-125a-5p inhibitor. (B) MM.1S and U266 cells were transfected with sh-BDNF-AS, or co-transfected with sh-BDNF-AS and miR-125b-5p inhibitor. The levels of cleaved caspase 3 (c-caspase 3), total caspase 3 (t-caspase 3), cleaved PARP (c-PARP) and total PARP (t-PARP) was evaluated by Western blot. Relative cleaved caspase-3 means cleaved caspase-3/total caspase-3, and relative cleaved PARP means cleaved PARP/total PARP. *p < 0.05, **p < 0.01. [file 12979_2021_258_MOESM3_ESM.tif]
